# An ecological approach to understanding transitions and tensions in complex learning contexts

**DOI:** 10.1038/s41539-024-00267-1

**Published:** 2024-09-06

**Authors:** Luke McCrone, Martyn Kingsbury

**Affiliations:** Centre for Higher Education Research and Scholarship, Imperial College London, London, UK

**Keywords:** Human behaviour, Education, Education

## Abstract

The move away from transmission-based lecturing toward a more student-centred active learning approach is well evidenced in STEM higher education. However, the examination of active learning has generally remained confined to formal timetabled contexts, with assumptions made that students independently manage the transition between timetabled and non-timetabled learning. This paper introduces research findings from a mixed methods study that used an ecological approach when investigating student transitions between a formal lecture theatre and adjacent informal breakout space in a UK STEM university. Using quantitative occupancy monitoring data to analyse usage patterns of both spaces, in combination with qualitative ethnographic observations and field interviews, permitted a purposeful exploration of student engagement with transitions within and between the two learning spaces. The ecological approach aided the discovery of spatial, pedagogic and agentic transitions and tensions, which subsequently informed strategic modification of space across the institution to facilitate the adoption of active learning pedagogy.

## Introduction

There is increasingly widespread recognition in UK higher education that traditional transmission lecturing is less effective than more student-centred active learning, particularly in science, technology, engineering, and mathematics (STEM) fields^1,2^. Active learning aligns with the social constructivist perspective^3^, where instructional activities require learners to actively construct knowledge and integrate it with existing knowledge and experiences^4^. A separate body of literature has investigated and evidenced the relationship between this learning activity and the role of physical space^5^. Traditional learning spaces like lecture theatres that are designed for transmission-based pedagogies present challenges for enabling more active pedagogies^6^. The development of active learning classrooms, for example, signals a gradual shift away from the spatial and pedagogic assumptions underpinning these traditional approaches, which have prevailed since the time they were used for ancient oral traditions^7^. However, much of the literature has focussed on active learning within formal timetabled contexts^2^, with assumptions made that students independently manage transitions across timetabled and non-timetabled learning^8^. Turning our attention to informal learning spaces^9^, particularly those spaces adjacent to formal classrooms like corridors^10^, can help us to develop a more holistic understanding of active learning and the challenges of transitioning in both space and pedagogic intent.

The institution researched in this paper has grappled with transforming its curriculum, pedagogy and built estate to achieve its strategic objectives linked to active learning, technology enhancement, and equality, diversity and inclusion. To gain insight into the impact of these strategic changes, the present research explored how students physically transition between timetabled and non-timetabled learning spaces^8^. However, the underlying institutional tension between promoting active pedagogies and teaching those pedagogies within traditional educational infrastructure, whilst not impossible, presented challenging complexity. This tension is particularly pertinent as pedagogies and spaces diversify with the emergence of hybrid, apprenticeship-based and lifelong learning and the evolution of spaces that blur boundaries such as makerspaces^11^. With ‘misunderstanding’ being considered one of three drivers of unsustainable development^12^, there is first a need to discard less appropriate models of how complex, dynamic systems such as universities truly work. Several scholars, such as Ronald Barnett^13^ and Ian Kinchin^14^, have consequently challenged the higher education sector to think more ecologically. By drawing from ‘ecology’, a biological term used to inspect the complexity of the interrelationship between organisms and their environment^16^, they argue that we stand a better chance of addressing important issues relating to sustainability, widening participation and lifelong learning while equipping students for rapidly changing future needs and challenges^12^.

Ecological principles have been applied to human social and mental activity since the 1970s^17^ and can be similarly drawn upon in this context: interconnectedness, systems thinking, resilience, continuous learning, sustainability, and biodiversity of people and ideas^15^. Ecological systems theory, for example, has broadened our understanding of human development, positing that individuals shape their own learning while simultaneously influencing other people and activities in both their immediate and more remotely connected environments^18,19^. This thinking has since extended to ‘ecological leadership’ to perceive leaders as members embedded and distributed within the ecosystem who can become aware of the institution’s ‘natural history’^14^. This contextual history is best uncovered using ethnography to retrieve a diversity of voices from different settings and to observe the activities and beliefs of those living in those settings^20^. The notion of an ‘ecological university’ therefore embraces the imagination and creativity of individual learners who transform ideas, relationships, materials, and themselves while supporting actors in gaining an understanding of their context, each other, and the epistemic environments in which they learn^21^. Common-pool resource theory posits that the collective management of tangible and intangible resources, such as physical and social spaces, can maximise sustainable output and foster a sense of ‘place’ in universities^22^. Providing opportunities and conditions in which students can cultivate a shared sense of ownership and agency in spaces therefore seems to be important^23^ and can be supported via participatory design-based approaches^10^ and other initiatives that distribute leadership^24^.

In setting out to study physical transitions between a lecture theatre and adjacent informal space, we soon realised from our initial data that students were navigating several transitions. Therefore, in this paper, ‘transition’ refers both to students moving between these physical spaces, as well as to changes in pedagogic intent, such as transitioning from teacher-centred didactic delivery to student-centred group-based learning. ‘Tensions’ represent the conflicting pressures that often drive or result from these changes. We therefore use the terms transition and tension in this paper to describe multiple phenomena. By understanding how students are engaging with these transitions and tensions between formal and adjacent spaces, this paper seeks to make a unique contribution to our understanding of active learning and pedagogic space. We argue that through the use of mixed methods^25^ and an ecological approach, the institution became more aware of these transitions and tensions within the context of its natural history^14^. The ecological approach has assisted in understanding the complex interaction between learning space, pedagogy and agency, as well as the conditions under which students can actively learn. This awareness has informed subsequent modifications to institutional spaces as part of a more sustainable, inclusive and productive learning ecology.

## Results

### Spatial transitions and tensions

**Fig. 1:**
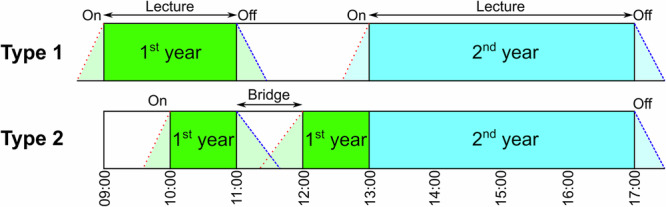
Lecture theatre type 1 and type 2 timetable configurations. Definition of type 1 and type 2 timetable configurations that were repeatable and analysable for the lecture theatre with on-ramp and off-ramp transitions labelled.

Lecture theatre and adjacent breakout space occupancy data were analysed in the context of the timetable to better understand the relative usage patterns before and after timetabled activity; the term ‘spatial’ is hence used here to describe the intersection between the physical spaces and the timetable. We defined two types of frequently recurring timetable configurations, type 1 and type 2 (see Fig. 1), to study transitions between the two spaces in different contexts. The nature of student engagement with and pedagogic potential of these transition periods in each timetable configuration was subsequently further investigated using a mixed methods approach. This approach generated data that informed the conceptualisation of these transition periods as on-ramp (space and time just before the timetabled lecture) and off-ramp (space and time just after the timetabled lecture) transitions, labelled in Fig. 1.

**Fig. 2:**
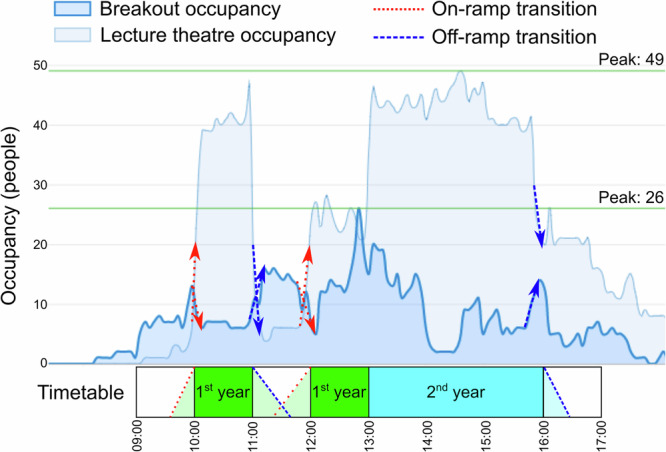
Occupancy plot for type 2 timetable configuration. Lecture theatre and breakout space occupancy plot labelled with type 2 timetable and occupancy changes as cohorts transition into (on-ramp) and out of (off-ramp) the lecture theatre.

The group of students ‘meaningfully’ engaging in on-ramp and/or off-ramp transitions was defined as the percentage of the incoming or outgoing lecture cohort who remained in the breakout space for longer than 5 min. These transitions are annotated as opposing arrows in Fig. 2 which displays a type 2 timetable occupancy profile to mark periods in which student cohorts seemed to enter or exit the lecture theatre and gather in the adjacent breakout space. The architectural divide between the two spaces resulted in predictable inflows and outflows of users that were able to be visualised using occupancy plots (like in Fig. 2).

Although our interest was primarily in students associated with the lecture cohort, the anonymity of the occupancy data meant that the identities of these students could only be inferred; ethnographic observations clarified the apparent identities and activities of users, while field interviews enabled us to confirm these demographic details for key instances. Ethnographic observation and interviews in the adjacent breakout space confirmed that, in general, most students were waiting and preparing for the timetabled lecture during the on-ramp transition period and reflecting and planning together during the off-ramp transition period. This qualitative data collection hence supported our characterisation of the breakout space as an area of pedagogical potential proximal to the lecture theatre. Being equipped with comfortable furniture and amenities better-supported students in their engagement with this potential during on-ramp and off-ramp transitions and associated learning activities.

The automated recording of occupancy data enabled the analysis of occupancy patterns across the expected, timetabled working week for 6 weeks with 14 days (120 h) data studied in more detail to confirm and further investigate the type 1 and type 2 timetable configurations. Subtracting lecture theatre and breakout space occupancy during timetable crossover periods provided data from which averages could be calculated for the percentage of year 1 or year 2 cohorts remaining and engaging with transitions in the breakout space. The average percentages provide insight into the potential influence of the timetable configuration on whether students were more or less likely to use the adjacent breakout space during transition periods. Based on the arithmetic mean, more (43%) of the students meaningfully engaged in the off-ramp transition during the ‘bridge’ space following the 1st year lecture in the type 2 timetable, whereas less (19%) of the students meaningfully engaged in the off-ramp transition in the type 1 timetable (see Fig. 1). The type 1 configuration seemed to provide less incentive for 1st year students to remain in the breakout space beyond their 9:00–11:00 lectures given that their timetabled expected interactions were not in that space. This was confirmed in field interviews, for example, a 1st year chemical engineering student was observed leaving her 10:00–11:00 chemistry lecture and explained how she typically used the breakout space:

“I usually tend to just quickly go over stuff, often with friends…purely because of its location and proximity to the lecture theatre”.

This behaviour of quickly reviewing material was mirrored during observation of other students leaving the same lecture 10 min prior, two of whom were overheard sharing “I kind of lost it after…” and “Did you understand…” as they stood to leave the lecture theatre just after the session had finished. While two students were observed asking the lecturer questions at the front of the lecture theatre, all but three immediately exited the lecture theatre and breakout space, likely heading to the campus library, to buy lunch or return to their hall accommodation.

In the type 1 timetable, 20% of the students meaningfully engaged in the on-ramp transition in the breakout space prior to their 2nd year lecture, whereas more (52%) of the 1st year students engaged in the on-ramp transition in the type 2 timetable (see Fig. 1). These statistics further support the potential of the ‘bridge’ period which might be understood as the interaction between the 1st year off-ramp and on-ramp transition periods in the type 2 timetable configuration. Ethnographic observation of student engagement within this bridge period revealed tensions between the expected timetabled teaching activity and more self-directed non-timetabled space and/or time. While occupancy analysis provided cohort-level insight into the spatial transitions within this bridge space and/or time (see Fig. 2 for example), observations aided in the discovery of less obvious complexities, such as tensions and potential within and between spaces and/or time. Field interviews with students deepened this understanding by confirming differences in student intent during this bridge time to either remain in the lecture theatre or breakout space ahead of the second lecture or migrate elsewhere to buy coffee or lunch, for example. Two 1st year chemical engineering students who departed their first lecture just before 11:00 (at the start of a bridge period) were observed moving together to a sofa in the breakout space. Overhearing their conversation, one admitted “The one thing I didn’t get…” to the other, who responded by confessing “How did he go from these three to these…” whilst pointing at paper notes. This observation strongly suggested that the two students were discussing difficult content from their fluid mechanics lecture, which was confirmed in a field interview in which they explained “Yes we were discussing concepts from the lecture” and that they “use this space between lectures”, stating their reason for using it as:

“It is purely because of the convenient location being by our lectures and that it is quite quiet. It is also quite spacious”.

The bridge period on this same day was dissimilarly used by a group of five 1st year students from the same cohort who chose to remain in the lecture theatre until around 11:26. Whilst they opted to use the lecture theatre instead of the breakout space, they seemed to engage in similar behaviour by clarifying misunderstandings with each other, before leaving the space ahead of their next lecture at 12:00. These findings suggest that the type 2 configuration may have the potential for more meaningful transition engagement than the type 1 configuration by providing 1st year students (who planned to attend both lectures) with a ‘bridge’ 11:00–12:00 containing 1 h of self-directed pedagogic potential sandwiched between 2 h of formal teaching contact.

Further understanding how these timetable configurations create the potential for connection and transition between timetabled activities might lead to alternative ways of informing timetable and instructional design and supporting active learning. While the mixed methods approach assisted in understanding transitions and tensions, a deeper understanding of this dynamic system eventually became limited by the language and concepts adopted. Taking an ecological approach helped us to overcome this limitation by enabling us to broaden the ethnographic focus on spatial transitions and tensions to also include those concerning pedagogy and agency. Furthermore, this approach crucially led to thinking about spaces not only as separate ecosystems but also as interconnected and integrated ecological zones within which there is different potential.

### Pedagogic transitions and tensions

Conceptualising the lecture theatre and breakout space as part of an interconnected ecological zone with on-ramp and off-ramp transitions provided an alternative way of thinking about the flows of people and information between the timetabled and non-timetabled periods. Ethnographically observing these transitions provided more nuanced insight into the tensions and student behaviours typical of these periods. The physical configuration, timetabled intention and historic usage of the lecture theatre created fixed patterns of expected behaviour, which centred around transmission-based approaches to teaching and learning where the teacher typically stands at the front and lectures to students who absorb information in the row-by-row seating area. The fixed power dynamic of the lecture theatre can often suppress student interaction and result in ‘failed’, hidden, and postponed pedagogic interactions^8^. ‘Failed’ pedagogic interactions are characterised by periods in which students with internal confusion feel disempowered and disinclined to raise their hand and ask questions, even when invited to do so by the lecturer. In instances where students ask a question, the lecturer may often simply provide an answer and, in so doing, maintain the expected flow of transmitted information and reinforce the power dynamic. While some lecturers occasionally deviate from this pedagogic style by, for example, encouraging class discussion using real-time surveys, they often feel limited by the inherent expectation of the physical space.

By choosing not to volunteer misunderstandings in front of the class during such ‘failed’ pedagogic interactions, some students can instead undergo hidden pedagogic interactions. These can be observed as brief periods of whispering and question exchange between neighbouring students or as students consulting personal technology during the lecture. They are termed ‘hidden’ because they do not explicitly conform to transmission-based pedagogic expectations and are often actively discouraged by the teacher who commands students’ attention. One lecturer was observed responding to this type of student interaction in a 1st year lecture by telling the whole class to “please listen carefully, this is extremely important” and further reinforcing the expected dynamic by responding to perceived distraction with “no talking please”.

**Fig. 3:**
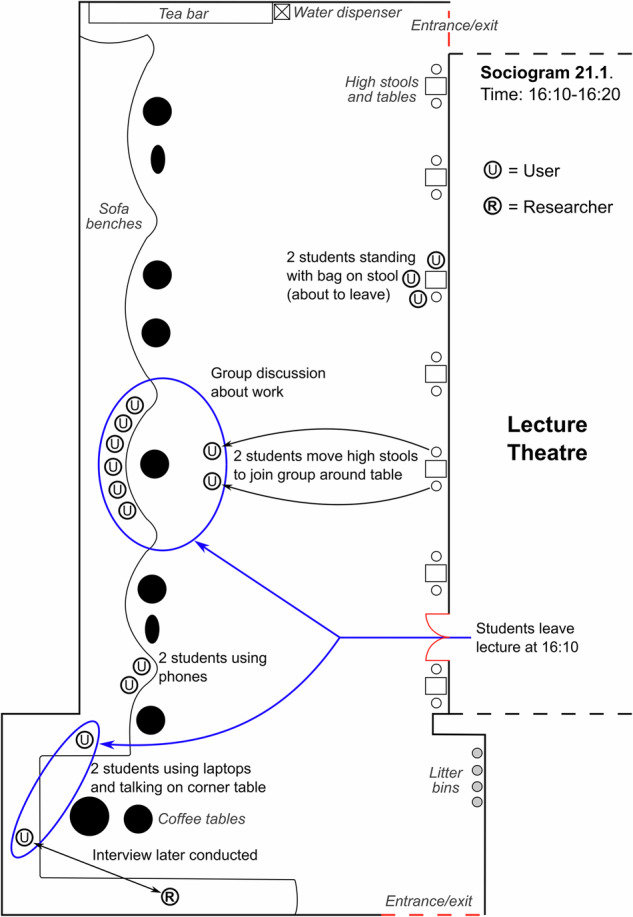
Sociogram of breakout space during off-ramp transition. Digitised sociogram of the breakout space showing user behaviour and interaction during an off-ramp transition immediately after a 2nd year lecture in the adjacent lecture theatre.

Other students may respond differently to their internal confusion by ‘postponing’ their questions to the end of the lecture. Some students undergoing these postponed pedagogic interactions during an off-ramp transition were observed gathering at the front of the lecture theatre to question the lecturer individually and more privately. Furthermore, the sociogram in Fig. 3 typifies group learning behaviour observed during an off-ramp transition period in the breakout space. In this case, the sociogram was recorded as soon as students left a 2nd year lecture timetabled until 16:00 to observe how members of the cohort engaged with the transition between the lecture theatre and adjacent space. The sociogram was recorded on a day configured with a type 2 timetable during an off-ramp transition much like the final one labelled on the occupancy plot in Fig. 2 (for a different day). As seen in the sociogram, a large group of eight students was observed engaging in a postponed pedagogic interaction in which they left the lecture theatre and immediately organised themselves around a table in the breakout space. The size of the group meant it was inappropriate to approach them for a field interview, yet their conversational tone and behaviour suggested they were discussing something work-related and possibly in relation to a “project”. The sociogram helped us to identify and approach a different chemical engineering student for a field interview in the corner of the breakout space who had also left the 2nd year lecture. She shared that she used the breakout space “because of its location” and that it is “quite comfortable because of the furniture”. This supports the view that the presence of such amenities and affordances outside of the lecture theatre can support students’ postponed pedagogic interactions.

As both teachers and students are subject to power dynamics and associated expectations that promote the transmission/absorption of information during timetabled learning, passive transmission-based learning is often perceived as a lower-risk activity than more student-centred active learning. In suppressing certain pedagogic potentials, a ‘pedagogic tension’ exists, within which students can sometimes manage the tension and more freely exercise their pedagogic intent. These pedagogic potentials were observed as ‘failed’, hidden and postponed pedagogic interactions, the behavioural signatures of which were visualised as sociograms (such as in Fig. 3) and confirmed using field interviews. Conceptualising this pedagogic tension between traditional passive learning and active learning as an ecological phenomenon changed how we thought about the space between the lecture theatre and the breakout space, while also recognising more broadly the opportunities for strategic changes in pedagogic mindset and behaviour.

### Agentic transitions and tensions

Investigating how agency and ‘ownership’ transitioned between the timetabled lecture theatre and non-timetabled breakout space and/or time periods aided in obtaining a better understanding of the conditions needed for inclusive active learning. Transmission-based teaching created pressure for students to experience ‘failed’, hidden and postponed pedagogic intent. On-ramp and off-ramp periods were ethnographically determined to be important spaces for supporting these transitions and managing tensions, as well as key sites of strategic change. Field interviews confirmed that students perceived less ownership of the lecture theatre than the teacher, who predominantly controlled the lecture interaction. Students also confirmed that they perceived the adjacent breakout area as a more democratic space, including one student who explained how they “can talk here and make some noise” whilst feeling “free to do anything”. In informal conversations, teachers concurred that they found it helpful to answer one-on-one student questions in a more informal setting like the breakout space.

The fixed power dynamic and potential fear of judgement often implicit in ‘typical’ lecture theatre interactions seemed to emphasise a more democratic power dynamic in the breakout space, such that students felt emancipated to direct questions and take more risks in their learning in this adjacent space. Student-directed questioning of peers and the teacher, as they physically moved from the lecture theatre into the breakout space during off-ramp transitions, suggested a renewed sense of agentic ownership in their learning. The affordances and flexibility of the breakout space meant that students under their own control could form pairs and small learning groups more easily than in the row-by-row lecture theatre. Ethnographic observations confirmed that this apparently incidental and procedural activity resulted in important dialogue and sharing between students, their peers and the teacher. The ‘agentic tension’ between the teacher-owned lecture theatre and the student-owned breakout space might be understood more broadly as an important tension between the historic way of learning traditionally in a lecture theatre and the pedagogic intent for learners to discover and explore via more active learning. Understanding this agentic tension also led to new ways of considering/managing the pedagogic balance between teacher-centred transmission and student-centred independent learning; this potential balance is dynamic and different for every student, cohort and teacher.

The public nature of the breakout space also led to the possibility of tensions between different user groups who perceived differing ownership of the space. These tensions changed throughout the academic year—based on who frequented the adjacent lecture theatre and surrounding spaces—and influenced the perceived agency of different users. The lecture theatre predominantly served early-year undergraduate students, who, as a function of their habitual ingress and egress via the breakout space, somewhat resulted in cohort familiarity and feelings of ownership; other research has shown that the intensity of these feelings can increase with the time spent there^26^. Studying on-ramp and off-ramp transitions and tensions between the two spaces provided insight into how different user groups interacted and vied for space ownership depending on their planned or unplanned activity. Later-year undergraduate students, for example, reported a change in their use of the breakout space (since their time as early-year undergraduates) based on their changed location of timetabled activities. This can be demonstrated by the field interview response of a 3rd year chemical engineering student who was interviewed with her friend in the breakout space:

“I like departmental spaces because of convenience and because of the micro community from being around other departmental members and friends. However, I would also say the way my friends and I use departmental spaces has changed over the course of our degree, as we now use different spaces to 1st year students, for example”.

Similarly, postgraduate students confessed to a greater sense of ownership during the summer months when undergraduate students no longer had timetabled teaching. This is exemplified by the interview response of a PhD student in chemical engineering who described the breakout space as:

“Not a place for work, because the undergraduates are here—sometimes I can’t even come here to eat because it is so full. Undergraduates tend to do group work here a lot as they have nowhere else.”

These agentic tensions therefore governed the sense of ownership different user groups felt in the space throughout the academic year and influenced how these users could enact certain activities. Framing these dynamic and temporally changing agentic tensions as ecological phenomena supported a deeper understanding of their nature and impact on student pedagogic interactions.

## Discussion

This paper has used data from a mixed methods study of student pedagogic interactions in a traditional lecture theatre and adjacent transitional space to illustrate the potential of an ecological approach, when investigating complex transitions and tensions between timetabled and non-timetabled learning spaces in a STEM university context. Three different categories of transitions and tensions between these spaces have been defined: ‘spatial transitions and tensions’, which exist as a function of timetables and architectural divides between formal and informal space and the associated expectation to learn in the formal timetabled space and/or time; ‘pedagogic transitions and tensions’, which exist between teacher-centred transmission intent within a space and student-centred intent, which can result in ‘failed’, hidden and postponed pedagogic interactions where students negotiate their own space for tackling misunderstanding; and ‘agentic transitions and tensions’, which occur between the fixed power dynamic of the formal lecture theatre and the more democratic nature of the adjacent informal space, resulting in differing agentic ownership between students and teachers in the different learning spaces.

Initially thinking about the lecture theatre and breakout space as fixed, isolated entities presented a barrier to understanding these complex interactions and tensions. We postulate that the ecological concept of the ‘ecotone’ may significantly advance our understanding of complex interactions between space and pedagogy in transition and tension. From an ecological perspective, ecotones not only represent zones of overlap and transition—for example, in estuarine intertidal zones between ocean and river ecosystems—but also exist as a distinct third ecosystem subject to conflicting tidal and river forces which create tensions and alter its position and makeup^27^. The increased biodiversity of edge species within an ecotone, which translates etymologically to mean ‘ecologies in tension’, emerges from the opportunities afforded by two ecosystems coexisting in an interconnected and dynamic way. By being less encumbered by the inertia and rules of the adjoining spaces, ecotones present opportunities for transition and potential evolutionary innovations that feedback into the core^28^. Although the use of the ecotone concept in higher education is limited^27,29^ and presents challenges by applying a metaphor from nature to model social systems^30^, its application in our research suggests its potential to transform our understanding of complex phenomena within evolving university systems.

Adopting an interrelated, dynamic view of the complex context of learning in a changing situation is necessary, given a rapidly changing context within which increasingly diverse models of learning can have a specific educational meaning. Although this paper has introduced the ecotone concept as a way of thinking about how space, pedagogy and agency interact and how tensions between them inhibit or enable learning, there may be other potential applications of the ecotone. For example, the concept could help us to think about a diverse group of students, each with individual cultural and learning expectations; different initial knowledge and skills; and different strategies, approaches and goals working together. Managing possible tensions within these groups while embracing a ‘biodiversity’ of people and ideas can become an important source of system resilience^31^. Perceiving this diversity as a source of positive pedagogic potential can inform the design of timetables and spaces for more productive active learning.

With the World Economic Forum anticipating the ‘metaverse’ as one of the top ten emerging technologies^32^, it is arguably important to think about how this new ‘in-between’ reality will enhance connectivity between people. For example, there has been a recent move away from dualistic definitions of online and physical space to ‘onlife space’^33^, which encapsulates both the physical and virtual realms and acknowledges the fundamental role of information technology in changing and activating physical spaces. The ecotone might be a credible metaphor for conceptually framing these onlife spaces, helping to acknowledge transitions and tensions that exist between reality and virtuality; the concept has separately been positioned as a useful metaphor for conceptualising the ‘…third space at the intersection of analogue, digital, and postdigital learning spaces’^29^.

With the institution’s desire to increase active learning pedagogy, our findings encouraged a reflection on the extent to which existing learning spaces and timetabled pedagogic interactions were always appropriate for students to navigate transitions between traditional transmission-based learning and more active learning. Applying the ecotone metaphor first helped with recognising the formal and informal spaces as greater than the sum of their parts, which facilitated a more nuanced data analysis and wider institutional discussion that raised awareness of tensions. It also subsequently evidenced the redesign of several lecture theatres, with the intent of providing spaces better designed to facilitate more flexible pedagogic and agentic transitions within both timetabled and non-timetabled periods. However, redesigning traditional lecture theatres to be more architecturally aligned with active learning classrooms^7^ is time- and cost-intensive and will mostly empower local changes to teacher and student agency. The already disproportionate capital investment into UK university education space, exceeded only by staff budgets, renders a broader redesign of all campus lecture theatres difficult^22^.

By instead using a mixed-method ecological approach to highlight the pedagogic potential of informal transitional spaces that exist between these more formal spaces and interactions, ecotone thinking can empower teachers to reconsider their use of existing spaces such as the lecture theatre and breakout space introduced in this paper. For instance, inviting students to move between such spaces can aid group formation and/or transitions between transmission-based lecture segments and more active group-based interactions. The understanding gained from these incidental and purposeful transitions between formal and informal spaces informed a series of student-staff partnership projects that redesigned ‘transitional spaces’ just outside of lecture theatres^10^. The ecological thinking therefore resulted in a ‘regenerative design’ approach^34^ in which spaces were redesigned more cost-effectively, teachers could evolve their perceived and actual use of space and students could better connect their timetabled learning to various contexts.

The sector-wide shift from an industrial model of universities towards an ecological model^14^ could itself be thought of as an ecotone within which students, teachers, policymakers, administrators and leaders operate and negotiate change. The ecotone concept may be valuable as a framework for considering complex, dynamic situations in which two or more things interact with the possibility of tensions existing between them. Higher education institutions that embrace an ecological mindset might be better prepared to navigate disruptions, whether they stem from technological advancements, shifts in student demographics, or global events such as the COVID-19 pandemic. The goal might be to move from a transactional to a transformative view of higher education^29^, which better supports the development of an ‘autonomous lifelong, life-wide learner, a capable knowledge worker, and a critical citizen’^24^ who has the capacity to change, learn and tackle the wicked challenges that the world faces.

## Methods

### Settings and participants

**Fig. 4:**
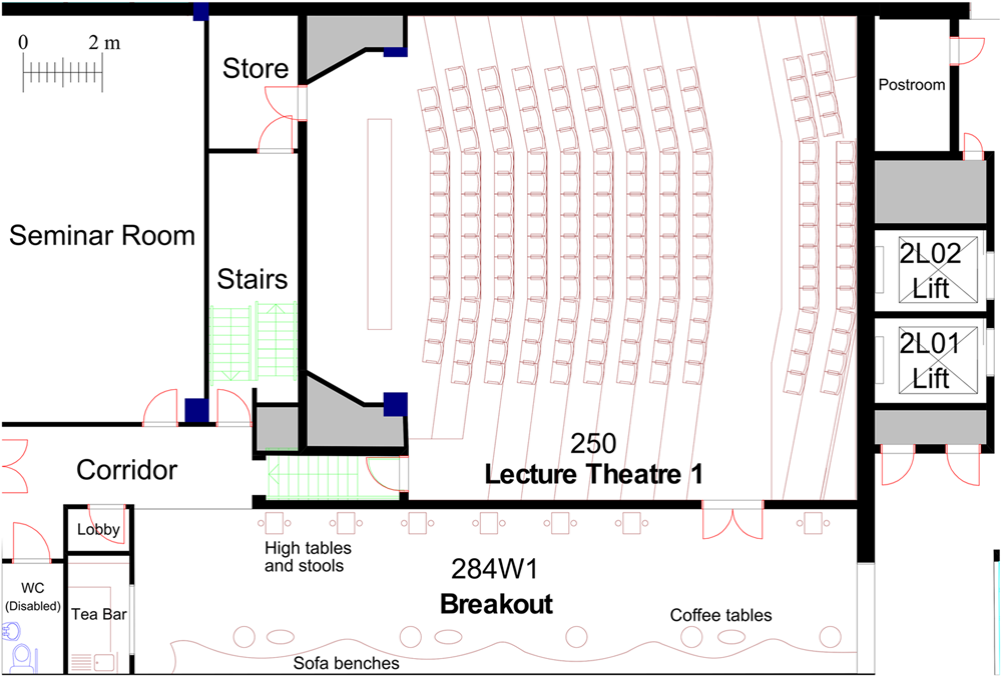
Floorplan diagram of breakout space and lecture theatre. Breakout space at the base shows a variety of furniture types and entrances to other spaces, including the traditional lecture theatre above with raked row-by-row seating for transmission-based teaching.

This research aimed to explore the nature of transitions within and between a ‘traditional’ lecture theatre used for timetabled activity and an adjacent informal breakout space (see Fig. 4 floorplan) in the institution’s chemical engineering department. This research setting was selected because of the interesting tension in activity between the lecture theatre—a row-by-row space with a 160-seat capacity serving 1st and 2nd year undergraduate classes of 30–150 students—and the public nature of the breakout space, used freely by a diverse group of users from inside and outside the department. This tension made the setting ideal for exploring transitions and potentials between different types of learning activities. The configuration and pedagogical use of the lecture theatre exemplified the ‘status quo’ across the STEM institution at the time of data collection. This provided a useful starting point for understanding existing learning behaviours and transitions amid the institution’s strategic transformation of learning space, curriculum and pedagogy. Furthermore, studying this setting was opportune following the institution’s investment in occupancy monitoring technology, which generated anonymous occupancy data for both spaces. The occupancy technology automatically collected data 24 h per day, 7 days per week, all year round allowing us to identify occupancy patterns and transitions and target specific days and times of interest. The wider, longer-term occupancy dataset was initially analysed at a higher, less granular level to select an appropriate setting for the research. Once the lecture theatre and breakout space had been selected, around 120 h of occupancy data spanning two academic years were analysed in more granular detail to investigate cohort transitions between the learning spaces, including for different timetable configurations.

This occupancy data informed our subsequent targeting of data collection resources when employing the qualitative methods^25^, enabling a more efficient and purposeful targeting of observations. We conducted 30 observations on 29 separate weekdays within 22 weeks across a period of 11 months. Given observations lasted between 30 min and 90 min, depending on if timetabled lecture sessions had also been observed, observations totalled around 24 h and were always conducted within the confines of the academic day (09:00–17:00) and term time. Straight after some observations, we also conducted a total of 21 field interviews with 25 student participants (as some interviews involved more than one participant).

Participants were purposefully sampled for field interviews based on their observed learning behaviours during transitions between timetabled and non-timetabled spaces and periods. As these field interviews were designed to be brief (5–10 min) and non-disruptive, we retrieved the degree type and year of study from participants, opting to note other demographic details based on our observation to avoid taking too much time. Of the 25 field interview participants, close to 50% were from the 1st and 2nd year undergraduate chemical engineering cohorts observed in lecture sessions; 4 interview participants were students from other departments and 4 participants were studying postgraduate degrees, demonstrating the user diversity of the breakout space. For a notional 40-h study week, the early-year undergraduate cohorts were expected to attend teaching contact for 25% of their time, with the remaining 75% of the time being spent independently studying. This emphasis on informal work is dictated by the degree assessment, which in the early years consisted of 10% practical, 20% coursework and 70% examination. The curricular requirement for formal and informal learning in this degree context made the transitions between timetabled and non-timetabled learning particularly interesting, and typical of many other STEM subjects taught at this institution.

### A mixed method for understanding transitions and tensions

The combination of quantitative space occupancy data with qualitative insights from ethnographic observations and field interviews led to a deeper understanding of how students perceive and engage with the learning spaces and the transitions between them^25^. The wider, longer-term occupancy dataset was initially analysed at a higher, less granular level for various spaces across the campus to identify potentially interesting settings in which qualitative data collection might be targeted; our secondary use of this occupancy data deviated from its intended institutional purpose of optimising room bookings and space efficiency. Occupancy insights for the lecture theatre and breakout space helped to reveal the ecological nature of the learning environment with inflow, outflow, transition and dwelling of people within and between the spaces. The occupancy data also guided the more resource-intensive targeting of ethnographic observations and field interviews which allowed us to interpret the meaning and nuance of those transitions and possible associated tensions.

‘Naturalistic’ ethnographic observation protocols^35^ were chosen because they are non-participant and minimise the chances of participants altering their behaviour; as a younger researcher, I was able to remain inconspicuous as an observer. Observations were recorded as field notes on a laptop and were sometimes supplemented with floorplan-based sociograms which captured person-person and person-space interactions within 10-min snapshots of breakout space activity (see Fig. 3 for example). Sociograms were mostly recorded during timetable transition periods (such as immediately after a lecture when students would flow into the breakout space) and helped us to target field interviews by visualising the broader context, identifying specific individuals or small groups who exhibited interesting learning behaviour whilst being sensitive to who we approached. Field interviews were initiated with verbal consent due to the informal low-risk nature of interviews, which posed structured questions pertaining to how exactly participants were using the space (as compared to the observation), how often they typically used the space, why they chose to use this specific space and where else they would typically carry out the same activity on campus. Informed consent was not used for these brief in situ field interviews, a decision approved as part of two detailed applications (reference numbers EERP1718-021 and EERP1819-012) made to Imperial College’s Education Ethics Review Process. The ethics committee agreed that using informed consent protocols for 5–10-min field interviews posed an inappropriate time cost for participants, whilst potentially affecting the quality of collected data by distancing it from the observed behaviour. Retrieving brief verbal consent posed less risk and was arguably more commensurate with the uncontentious informal nature of questioning.

Thematic analysis^36^ was used to analyse the ethnographic observation and field interview data by developing ‘themes’ in the qualitative data. These themes are patterns of shared meaning unified by a central organising concept, which emerge inductively from drawing relationships between different segments of the data that are assigned smaller units of meaning called codes. Analysis began during the collection of ethnographic field notes given these inscriptions were shaped by what I ‘saw’ and the choices I made as an ethnographer. To minimise bias, we remained reflexive and used our positioning and contextual knowledge, including when looking at patterns in the objective occupancy data or when using those results to target subsequent observations, to repeatedly make meaning of the data and develop a critical perspective^20^. Observation and field interview data were separately analysed in NVivo software to develop codes and themes which could then be understood holistically.

The mixed method approach and analysis of that data embodied ecological principles by dynamically and reflexively moving between remote analysis of cohort-level occupancy patterns and in situ observations and field interviews. This triangulation supported a more complete understanding of phenomena and a more resourceful collection of authentic data. The use of this approach in other settings in the institution has informed the development of infrastructure, spaces and places in which transitions and tensions are better managed and learning ecologies are able to organically develop.

### Reporting summary

Further information on research design is available in the Nature Research Reporting Summary linked to this article.

## Supplementary information

Editorial policy checklist

Reporting summary

## Data Availability

Some of the data collected in the research study is available, as limited by ethical approval, and can be requested by contacting the corresponding author (L.M.).
